# Retrospective study of canine cutaneous tumors submitted to a diagnostic pathology laboratory in Northern Portugal (2014–2020)

**DOI:** 10.1186/s40575-022-00113-w

**Published:** 2022-02-25

**Authors:** Ana Luísa Martins, Ana Canadas-Sousa, João R. Mesquita, Patrícia Dias-Pereira, Irina Amorim, Fátima Gärtner

**Affiliations:** 1 Instituto de Ciências Biomédicas Abel Salazar, Universidade Do Porto (ICBAS), 4050-313 Porto, Portugal; 2 Epidemiology Research Unit (EPIUnit), Instituto de Saúde Pública da Universidade Do Porto, Porto, Portugal; 3 Instituto de Investigação E Inovação Em Saúde da Universidade Do Porto (i3S), 4200-135 Porto, Portugal; 4 Instituto de Patologia E Imunologia Molecular da Universidade Do Porto (IPATIMUP), Porto, Portugal; 5 Faculdade de Ciências, Universidade Do Porto (FCUP), 4169-007 Porto, Portugal

**Keywords:** Cutaneous tumor, Cancer, Dog, Breed, Anatomic location, Sex, Age

## Abstract

**Background:**

Cutaneous neoplastic diseases are the most and second-most frequently reported tumors in male and female dogs, respectively. The aims of this study were to report the occurrence of canine cutaneous tumors in a pathology laboratory located in Northern Portugal between 2014 and 2020, and to characterize and categorize the anatomical locations, breed, age, and sex of the animals affected with different types of neoplasms.

**Results:**

Throughout the 7-year study, 1,185 cases were diagnosed as cutaneous tumors, with 62.9% being classified as benign, and 37.1% as malignant. Mast cell tumors (22.7%) were the most frequently diagnosed tumor type, followed by benign soft tissue tumors (9.7%), sebaceous gland tumors (8.1%), vascular tumors (7.9%) and soft tissue sarcomas (7.6%). Cutaneous tumors commonly exhibited multicentric occurrence (14.6%) followed by single occurrence in hindlimb (12.1%), forelimb (8.6%), buttock (7.1%), abdominal (6.5%) and costal (5.2%) areas. The odds of developing cutaneous neoplasia were higher with increasing age (*p* < 0.001). Females had an increased odds of developing skin tumors compared to males (crude OR = 2.99, 95% (2.51, 3.55); adj OR = 2.93, 95% (2.46, 3.49). Purebred dogs, as a group, showed a reduced odds of developing cutaneous tumors when compared to mixed-breed dogs (crude OR = 0.63, 95% (0.53, 0.74); adj OR = 0.75, 95% (0.62, 0.89).

**Conclusions:**

Mast cell tumors, benign soft tissue tumors and sebaceous tumors were the most common histotypes encountered. The epidemiological survey achieved with this study demonstrates the relative frequency of different types of tumors in this particular population. Furthermore, the results herein achieved can act as a basis or a beneficial reference for local veterinarians helping in the establishment of a preliminary and presumptive diagnosis of canine cutaneous tumors histotypes.

**Plain English summary:**

Skin tumors are the most and second-most frequently reported tumors in male and female dogs, respectively. The aim of this study was to report the occurrence of canine skin tumors in a diagnostic pathology laboratory located in Northern Portugal, between 2014–2020 and to characterize the anatomical distributions, breed, age, and sex of the animals affected by different skin tumors.

During this period, 1,185 cases were diagnosed as skin tumors; 62.9% were diagnosed as benign, while 37.1% were malignant. Mast cell tumors (22.7%) were the most frequently diagnosed neoplasia, followed by benign soft tissue tumors (9.7%), sebaceous gland tumors (8.1%), vascular tumors (7.9%) and soft tissue sarcomas (7.6%). Skin tumors commonly developed in more than one location (14.6%) followed by solitary development in hindlimb (12.1%), forelimb (8.6%), buttock (7.1%), abdominal (6.5%) and costal (5.2%) areas. An increased odds of developing skin neoplasms as the patient’s age increase was detected. Females showed an increased odds in comparison to male dogs. Purebred dogs presented decreased odds for developing skin tumors in comparison to mixed-breed dogs.

The information relevance achieved with this study demonstrates the relative frequency of different types of tumors in this particular population, acting as a basis or a beneficial reference for regional veterinarians when providing an initial diagnosis of canine skin tumors.

**Supplementary Information:**

The online version contains supplementary material available at 10.1186/s40575-022-00113-w.

## Background

A broad range of neoplasias can be found in the skin, subcutis and adnexa [1]. Skin tumors are amongst the most frequent canine tumors submitted for histopathological diagnosis. Since they are easily visualized by the owner, they are frequently brought to the attention of the veterinarian [2]. In male and female dogs, cutaneous neoplasias are the most and second-most frequently reported tumors, respectively [3–7]. A skin tumor diagnosis typically comprises cellular evaluation through cytology and histopathology and grading and search for metastasis for further clinical staging. In the great majority of cases, the preferred treatment for cutaneous tumors remains surgical excision. However, this decision depends on the type of neoplasm as well as its grade, stage, and location [1, 8]. In malignant tumors, radiation or chemotherapy can be used either as isolated or as adjuvant treatment.

Numerous retrospective studies have been performed to investigate canine cutaneous tumors (CCT) epidemiology [1, 8–11]. However, data compilation, study population, inclusion criteria, sample size, geographical regions and outcomes often vary between studies. In retrospectives studies, tumor incidence could be calculated based on population data obtained from national canine cancer registries and veterinary authorities or, in some cases, from information provided by diagnostic laboratories[1, 8–12]. Despite the differences in data collection and study population, all these investigations share the mutual aim of expanding knowledge about the occurrence of CCT.

Canine CT epidemiological data is limited worldwide and, according with the different geographic locations, distinct conditions such as breed preferences, environmental influences, living conditions and practices can significantly vary and influence the outcomes and conclusions of these studies [8, 13]. The first aim of this study was to report the occurrence of CCT based in case submissions to a diagnostic pathology laboratory in Northern Portugal, from 2014 to 2020. The second aim was to characterize and categorize CCT anatomical distributions, reporting breed, age, and sex differences. These neoplasms are responsible for considerable animal morbidity and veterinary health services pursue due to their high frequency. Therefore, knowledge of the exact incidence remains important, when planning, veterinary health policies.

## Methods

### Study population

From January 2014 to June 2020, tissue biopsies from 2,291 dogs were submitted to the Laboratory of Veterinary Pathology of Institute of Biomedical Sciences Abel Salazar (ICBAS Pathology Lab) for histopathological examination. This laboratory is based at the University of Porto and receives submissions from clinics located in Northern and Central Portugal, and to a lesser extent in Southern Portugal, namely Azores and Madeira islands. Surgical biopsies of CCT diagnosed during this time interval were selected from the laboratory database and details such as breed, age, and sex were recorded. Exclusion criteria included cases with more than one missing clinical information (*e.g.*, sex, age, breed, or anatomical location). Cases diagnosed as epithelial cyst or modified sebaceous and apocrine gland, such as ceruminous gland tumor, mammary gland tumor, anal sac gland tumor and meibomian gland tumor, were excluded in order to keep the focus on cutaneous tumors that may arise from any haired skin region. A tumor type with multicentric development was regarded as a single tumor event. The simultaneous manifestation of different tumor types and tumor recurrence in a patient were considered as separate multiple events [8]. The relative frequency (% of all cutaneous tumor types), occurrence rate (% of the total canine population in the database during the study period), and occurrence within breed of cutaneous tumors (% of the total specific breed population in the database during the study period) were calculated. Anatomical sites were labeled as cranial, facial, ear, neck, shoulder, pectoral, costal, dorsal, pelvic, buttock, tail, forelimb, forepaw, hindlimb, hindpaw, perigenital area, multicentric, and skin not otherwise specified (NOS) when the precise site of tumor development was not known. Anatomical location was recorded as multicentric when a particular tumor type (of the same grade, when applicable) developed in multiple (two or more) cutaneous regions. In mast cell tumors (MCTs), when no histological grading or no subclassification was attributed (subcutaneous or cutaneous), the term “not otherwise classified” (NOC) was used.

### Tumor diagnosis and classification

The ICBAS Pathology Lab archives comprises cases of canine neoplasias classified according to the World Health Organization (WHO) International Histological Classification of Tumors of Domestic Animal [14–16], evaluated by four veterinary pathologists, one of which is a Diplomate of the European College of Veterinary Pathology (FG). Both cutaneous and subcutaneous MCTs were analyzed independently since the existing histologic grading scheme should not be applied to subcutaneous forms [17, 21]. Additionally, MCTs were graded according to the Kiupel’s 2-tier grading system and Patnaik classification system [13, 17].

### Statistical analysis

To assess the influence of age, sex, and breed in the development of CT, binary logistic regression analysis, integrated with likelihood ratio test, was performed according to previous studies [8]. The analysis was performed using the Epicalc package of R software (R4.03). Dependent variable was defined as cutaneous tumor development (yes/no). Factors were defined as sex (reference = male), breed (reference = mixed breed), and age (reference = youngest quartile, 0–8 years). Since our study population in terms of age was not equally distributed across all groups, for the factor age, quartiles were established dividing the population into 4 consecutive quartiles: from 0–8 years; 8–10 years-old; 10–12 years-old and 12–19 years, with the “younger” group (0–8 years) taken as the reference group. For the analysis of the three factors (age, sex, and breed) two different models were performed: 1) one consisting in comparing age, sex and purebred vs mixed breed; 2) and the other model discriminated all specific breeds. Results were reported as odds ratios (OR) with its associated confidence interval (CI). A P-value of less than 0.05 was considered statistically significant. Two values of OR were used: crude OR and adjusted OR. The crude OR is obtained when considering the effect of only one predictor variable and only one independent variable in the model. The adjusted OR measure the association between a confounding variable, the outcome and controls for that value, providing an idea of the dynamics between predictors. Quartile assessment was only used for the risk analysis calculation and not for the descriptive statistical analysis. HFI (higher frequency interval) in the descriptive statistical analysis refers to the age range in which there was a greater number of diagnosed cases.

## Results

### Canine population in the database, 2014–2020

In this period, the database comprised a total of 2,291 dogs with a median age of 9 years (range = 0–20). Female to male ratio was 1:0.70 (female 58.8% and male 41.2%). The most common breeds were mixed-breed dogs (39.6%), Labrador retriever (13.7%), boxer (5.9%), German shepherd (3.9%), Yorkshire terrier (3.3%), poodle (3.1%), golden retriever (2.3%), cocker spaniel (2.1%), pinscher (2.0%), French bulldog (1.8%), beagle (1.4%), pit bull (1.2%), Rottweiler (1.1%), Siberian husky (1.0%) and Estrela mountain dog (0.9%) (Table S1).

### Study population

Based on the established criteria, during the 7-year study period, 1185 submissions were retrieved from 937 dogs in the database, other pathologies unrelated to skin tumors. Two or more tumor types were diagnosed in 162 dogs. The median age of the affected animals at the time of diagnosis was 10 years old (range = 0–18). Female dogs were more affected (51.4%) compared to males (48.6%). Among the 78 dog breeds with CT, the most affected were mixed-breed dogs (34.1%), Labrador retriever (18.1%), boxer (9.8%), cocker spaniel (3.5%), golden retriever (3.3%), German shepherd (2.5%), Yorkshire terrier (1.7%), pit bull (1.7%), poodle (1.5%) and French bulldog (1.5%), accounting for 77.6% of the total case.

### Anatomical distribution of lesions

**Fig. 1:**
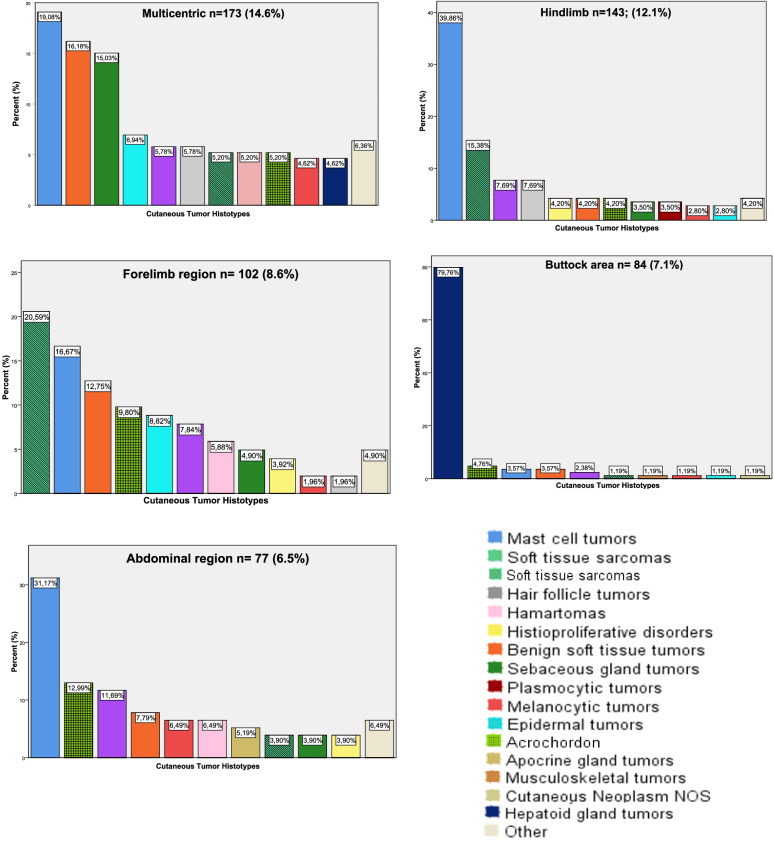
Five most common anatomical locations of CCT (*n* = numbers of tumors) and the relative frequency (%) of the most encountered tumor histotypes in each location

The anatomical sites where CCT frequently developed, as well as the ten most diagnosed tumor types are depicted in Fig. 1. Skin tumors were mainly found on the hindlimb (12.1%), forelimb (8.6%), buttock (7.1%) and abdominal (6.5%) areas, with more than 60 cases recorded in each of those body regions. Multicentric development was found in 14.6% of the cutaneous tumor cases.

### Tumor types

The relative frequency and occurrence rate of CCT (calculated based on the total canine population of the database, 2014–2020) is depicted in Table 1. In our database, CCT had an occurrence rate of 51.7%. Of the 1,185 cases, 62.9% were diagnosed as benign, while 37.1% were malignant. Broadly, MCTs were the most frequently diagnosed, followed by benign soft tissue tumors, sebaceous gland tumors, vascular tumors and soft tissue sarcomas. Breed, age, sex, and region distribution of these patients according with tumor histotype is summarized in Table 2. Age distribution in tumor type is depicted in Figure S1.

**Table 1:** Histopathological diagnosis, relative frequency, and occurrence rate of *CCT* recorded in the database of ICBAS Pathology Lab (total population = 2,291 dogs)

DIAGNOSIS	NUMBER OF CASES	RELATIVE FREQUENCY (% OF ALL SKIN TUMORS)
**EPITHELIAL TUMORS**	339	28.61
***EPIDERMAL TUMORS***	60	5.06
BASAL CELL CARCINOMA	6	0.51
PAPILLOMA	22	1.86
SQUAMOUS CELL CARCINOMA	32	2.70
***HAIR FOLLICLE TUMORS***	69	5.82
INFUNDIBULAR KERATINIZING ACANTHOMA	10	0.84
TRICHOLEMMOMA	2	0.17
TRICHOBLASTOMA	20	1.69
TRICHOEPITHELIOMA	27	2.28
PILOMATRICOMA	6	0.51
SUBUNGUAL KERATOACANTHOMA	3	0.25
***SEBACEOUS GLAND TUMORS***	96	8.10
SEBACEOUS ADENOMA	62	5.23
SEBACEOUS EPITHELIOMA	34	2.87
***APOCRINE GLAND TUMORS***	18	1.52
APOCRINE ADENOMA	8	0.68
APOCRINE CARCINOMA	6	0.51
***HEPATOID GLAND TUMORS***	88	7.43
HEPATOID ADENOMA	57	4.81
HEPATOID EPITHELIOMA	14	1.18
HEPATOID CARCINOMA	10	0.84
HEPATOID NEOPLASM	7	0.59
***EPITHELIAL TUMORS NOS***	8	0.68
ADENOCARCINOMA	1	0.08
CARCINOMA NOS	4	0.34
**MELANOCYTIC TUMORS**	43	3.63
***MELANOCYTOMA***	23	1.94
***MELANOMA***	20	1.69
**MESENCHYMAL TUMORS**	311	26.24
***BENIGN SOFT TISSUE TUMORS***	115	9.70
LIPOMA	86	7.26
INFILTRATIVE LIPOMA	6	6.98
FIBROMA	25	2.11
MYXOMA	3	0.25
***SOFT TISSUE SARCOMAS***	90	7.59
FIBROSARCOMA	2	0.17
PERIVASCULAR WALL TUMOR	20	1.69
PERIPHERAL NERVE SHEATH TUMOR	39	3.29
LIPOSARCOMA	2	0.17
SARCOMA NOS	21	1.77
SINOVIAL SARCOMA	3	0.25
MYXOSARCOMA	3	0.25
***VASCULAR TUMORS***	94	7.93
HEMANGIOMA	68	5.74
LYMPHANGIOMA	3	0.25
HEMANGIOSARCOMA	23	1.94
***MUSCULOSKELETAL TUMORS***	8	0.68
OSTEOSARCOMA	4	0.34
***MESENCHYMAL NEOPLASM NOS***	3	0.25
**HEMOLYMPHATIC TUMORS**	357	30.13
***MAST CELL TUMORS***	269	22.70
MAST CELL TUMORS NOC	4	0.34
CUTANEOUS MAST CELL TUMORS	244	20.59
SUBCUTANEOUS	21	1.77
***PLASMOCYTIC TUMORS***	18	1.52
PLASMOCYTOMA	17	1.43
***LYMPHOMAS***	6	0.51
T- CELL LYMPHOMA	2	33.33
***HISTIOPROLIFERATIVE DISORDERS***	61	5.15
HISTIOCYTOMA	58	4.89
HISTIOCYTIC SARCOMA	3	0.25
***ROUND CELL NEOPLASM NOS***	3	0.25
**HAMARTOMAS**	56	4.73
***HAMARTOMA***	56	4.73
**TUMOR LIKE LESIONS**	71	5.99
***ACROCHORDON***	71	5.99
**CUTANEOUS NEOPLASM NOS**	8	0.68
UNDIFFERENTIATED MALIGNANCY	8	0.68

*NOS*—not otherwise specified

*NOC*—not otherwise classified

**Table 2:** Breed, age, female to male ratio and frequent locations of the most common cutaneous tumor types. HFI—Higher frequency interval (age interval with the highest frequency for a specific tumor histotype). The number 0 means less than 1 year-old

Tumor histotypes (*n* = number of cases)	3 most affected breeds				Age (years)	Female to male ratio	Location (region)
	**Breed**	**n**	**% of all affected breeds**	**Occurrence within breed, %**			
**Mast cell tumor** **(*n* = 269)**	Labrador retriever	72	26.77	22.86	Range = 1–16 Mode = 10 Median = 9 HFI = 7–11	1: 0.81	Hindlimb (21.2%) Multicentric (12.3%) Abdominal (8.9%)
	Mixed	69	25.65	7.60			
	Boxer	41	15.24	30.15			
*Cutaneous mast cell tumor* *(n* = *244)*	Labrador retriever	67	27.46	21.27	Range = 1–16 Mode = 10 Median = 9 HFI = 7–11	1: 0.83	Hindlimb (20.5%) Multicentric (12.3%) Abdominal (8.6%)
	Mixed	64	26.23	7.05			
	Boxer	37	15.16	27.21			
**Benign soft tissue tumor** **(*n* = 115)**	Mixed	55	47.83	6.06	Range = 0–17 Mode = 10 Median = 10 HFI = 7–12	1: 0.42	Multicentric (24.4%) Forelimb (11.3%) Pectoral (10.4%)
	Labrador retriever	27	23.48	8.57			
	golden retriever	3	2.61	5.77			
*Lipomas* *(n* = *86)*	Mixed	41	47.67	4.52	Range = 1–17 Mode = 10 Median = 10 HFI = 8–12	1: 0.39	Multicentric (31.4%) Pectoral (11.6%) Forelimb/Costal (10.5%)
	Labrador retriever	21	24.42	6.67			
	golden retriever	3	3.49	5.77			
*Fibromas* *(n* = *25)*	Mixed	11	44.00	1,21	Range = 0–13 Mode = 7 Median = 8.5 HFI = 7–13	1: 0.56	Abdominal (20.0%) Forelimb (12.0%) Dorsal (8.0%)
	Labrador retriever	5	20.00	1,59			
	poodle/Rottweiler^#^	2	8.00	2,86/8.00			
**Sebaceous gland tumor** **(*n* = 96)**	Mixed	24	25.00	2.64	Range = 4—18 Mode = 13 Median = 12 HFI = 10–14	1: 0.88	Multicentric (27.1%) Neck (9.4%) Ear (8.3%)
	Labrador retriever	23	23.96	7.30			
	cocker spaniel	16	16.67	34.04			
*Sebaceous adenoma* *(n* = *62)*	Mixed	18	29.03	1.98	Range = 4–18 Mode = 13 Median = 12 HFI = 11–14	1: 0.72	Multicentric (33.87%) Neck (8.1%) Cranial (6.5%)
	Labrador retriever	11	17.74	3.49			
	cocker spaniel	8	12.90	17.02			
*Sebaceous epithelioma* *(n* = *34)*	Labrador retriever	12	35.29	3.81	Range = 7–17 Mode = 10/12 Median = 12 HFI = 10–14	1: 1.27	Ear (23.5%) Multicentric (14.7%) Facial (11.8%)
	cocker spaniel	8	23.53	17.02			
	Mixed	6	17.65	0.66			
**Vascular tumor** **(*n* = 94)**	Mixed	33	35.11	3.63	Range = 3–14 Mode = 7/8 Median = 8.5 HFI = 6–11	1: 0.92	Hindlimb (11.7%) Multicentric (10.6%) Abdominal (9.6%)
	Labrador retriever	15	15.96	4.76			
	Boxer	13	13.83	9.56			
*Hemangioma* *(n* = *68)*	Mixed	20	29.41	2.20	Range = 3–14 Mode = 7/10 Median = 9 HFI = 6–10	1: 0.94	Hindlimb (13.2%) Abdominal (13.2%) Forelimb/Dorsal (10.3%)
	Labrador retriever	13	19.12	4.13			
	Boxer	12	17.65	8.82			
*Hemangiosarcoma* *(n* = *23)*	Mixed	11	47.83	1.21	Range = 4–14 Mode = 8 Median = 8 HFI = 6–11	1: 0.77	Perigenital (21.7%) Multicentric (17.4%) Abdominal (8.7%)
	Dogo Argentino	2	8.70	28.57			
	German shepherd/pit bull^#^	2	8.70	2.22/7.14			
**Soft tissue sarcomas** **(*n* = 90)**	Mixed	41	45.56	4.52	Range = 2–18 Mode = 10 Median = 10 HFI = 9–12	1: 0.96	Hindlimb (24.4%) Forelimb (23.3%) Multicentric (10.0%)
	Labrador retriever	11	12.22	3.49			
	Boxer	9	10.00	6.62			
*Peripheral nerve sheath tumor* *(n* = *39)*	Mixed	19	48.72	2.09	Range = 2–18 Mode = 12 Median = 10 HFI = 10–13	1: 0.86	Forelimb (23.1%) Hindlimb (20.5%) Multicentric (10.3%)
	Boxer	4	10.26	2.94			
	German shepherd	4	10.26	4.44			
*Perivascular wall tumor (n* = *20)*	Mixed	11	55.00	1.21	Range = 2–15 Mode = 9/11/12 Median = 10 HFI = 9–12	1: 0.82	Hindlimb (40.0%) Forelimb (25.0%) Dorsal (10.0%)
	Boxer	4	20.00	2.94			
	Boerboel	1	5.00	100.00*			
**Hepatoid gland tumor** **(*n* = 88)**	Mixed	46	52.27	5.07	Range = 2–18 Mode = 9/10/11 Median = 11 HFI = 9–14	1: 6.33	Buttock area (76.1%) Multicentric (9.1%) Tail (6.8%)
	Labrador retriever	6	6.82	1.90			
	Estrela mountain Dog	4	4.55	20.00			
*Hepatoid adenoma* *(n* = *57)*	Mixed	30	52.63	3.30	Range = 2–18 Mode = 9 Median = 10 HFI = 9–14	1: 4.18	Buttock (75.4%) Tail (8.8%) Multicentric (7.0%)
	Labrador retriever	5	8.77	1.59			
	cocker spaniel	3	5.26	6.38			
**Tumor like lesions (*n* = 71)** *Acrochordon*	Boxer	15	21.13	11.03	Range = 1–17 Mode = 11 Median = 9 HFI = 7–11	1: 0.82	Abdominal (14.1%) Forelimb (14.1%) Multicentric (12.7%)
	Mixed	14	19.72	1.54			
	Labrador retriever	10	14.08	3.17			
**Hair follicle tumor** **(*n* = 69)**	Mixed	29	42.03	3.19	Range = 2–18 Mode = 6 Median = 8 HFI = 6–9	1: 1.16	Hindlimb (15.9%) Dorsal (14.5%) Multicentric (14.5%)
	German shepherd	7	10.14	7.78			
	bassett hound	5	7.25	38.46			
*Trichoepithelioma* *(n* = *28)*	Mixed	16	57.14	1.76	Range = 4–14 Mode = 6 Median = 7 HFI = 5–11	1: 0.87	Multicentric (25.0%) Dorsal (17.9%) Hindlimb (14.3%)
	bassett hound	2	7.14	15.38			
	boxer/golden retriever^#^	2	7.14	1.47/3.85			
*Trichoblastoma* *(n* = *20)*	Mixed	4	20.00	0,44	Range = 2–13 Mode = 9/13 Median = 9 HFI = 9–13		Cranial (30.0%) Ear (15.0%) Facial (15.0%)
	German shepherd	3	15.00	3,33		1: 3.00	
	cocker spaniel	2	10.00	4,26			
**Histioproliferative disorders** **(*n* = 61)**	Mixed	20	32.79	2.20	Range = 0–14 Mode = 1 Median = 2 HFI = 0–3	1: 0.85	Ear (16.4%) Facial (11.5%) Pectoral (11.5%)
	Boxer	10	16.39	7.35			
	French bulldog	7	11.48	17.07			
*Histiocytoma* *(n* = *58)*	Mixed	20	34.48	2.20	Range = 0–14 Mode = 1 Median = 1 HFI = 0–3	1: 1.07	Ear (17.2%) Facial (12.1%) Pectoral (12.1%)
	Boxer	9	15.52	6.62			
	French bulldog	7	12.07	17.07			
**Hamartomas (*n* = 56)**	Mixed	18	32.14	1.98	Range = 3–18 Mode = 7/11 Median = 10 HFI = 7–12	1: 0.75	Multicentric (16.1%) Forelimb (10.7%) Abdominal (8.9%)
	Labrador retriever	13	23.21	4.13			
	Boxer	12	21.43	8.82			
**Epidermal tumor** **(*n* = 60)**	Mixed	14	23.33	1.54	Range = 3–17 Mode = 8/9/10 Median = 9 HFI = 7–10	1: 1.22	Multicentric (20.0%) Forelimb (15.0%) Facial (8.3%)
	Labrador retriever	12	20.00	3.81			
	Dogo Argentino	6	10.00	85.71			
*Squamous cell carcinoma* *(n* = *32)*	Mixed	8	25.00	0.88	Range = 3–15 Mode = 9/10 Median = 10 HFI = 9–12	1: 1.13	Multicentric (31.3%) Facial (9.4%) Forepaw (9.4%)
	Dogo Argentino	5	15.63	71.43			
	Labrador retriever	4	12.50	1.27			

^*^This breed only had one exemplar in our database which result in 100% of breed specific occurrence

^#^both breeds present the same number of cases and frequencies, although the occurrence within breed is different

Mast cell tumors were categorized into cutaneous (90.7% of all MCT cases), subcutaneous (7.8%) and NOC (1.5%). MCTs had a higher number of cases concentrated in older dogs and were located in the hindlimb, abdominal or costal region (8.6%) and 12.3% presented multicentric development. Labrador retrievers, mixed-breed dogs and boxers were the most affected. Labrador retriever and boxers had a higher breed-specific occurrence than the other breeds.

In this study, two classification systems were used for cutaneous MCT, namely: Patnaik [13] and/or Kiupel [17]. According to Kiupel classification system, 111 cases were classified as low-grade, and 45 considered high-grade (88 cases were not subjected to this classification). With Patnaik’s system, 29 cases were classified as grade I, 146 cases as grade II and 50 cases as grade III (19 cases were not subjected to this classification).

In boxers, Patnaik’s grade I and grade II (28.06% and 62.86%, respectively) were the most observed, whereas with Kiupel’s classification system, low grade cases had the highest frequency (84.0%). In mixed breed dogs, the most common classification grade was Patnaik grade II (64.40%), however regarding Kiupel’s classification, low grade cases were the most common for this breed (66.67%). Similarly to mixed breeds, Labrador retrievers had a higher number of cases classified as Patnaik’s grade II and grade III (67.21% and 19.67%, respectively) and as low-grade (78.57%) according with Kiupel’s. All the details of the frequency distribution based on both classification systems for the three most common breeds with cutaneous MCTs is compiled in Table 3.

**Table 3:** Frequency distribution of the Patnaik’s classification system and Kiupel’s classification system in the three breeds with the highest occurrence of cutaneous mast cell tumor

BREEDS	BOXER		MIXED BREED		LABRADOR RETRIEVER		ALL BREEDS**
	**n**	**%**	**n**	**%**	**n**	**%**	**n**
**PATNAIK’S CLASSIFICATION SYSTEM**							
GRADE I	10	28.06	4	6.78	8	13.11	29
GRADE II	22	62.86	38	64.40	41	67.21	146
GRADE III	3	8.57	17	28.81	12	19.67	50
NSC*	2		5		6		19
**KIUPEL’S CLASSIFICATION SYSTEM**							
LOW GRADE	21	84.0	28	66.67	33	78.57	111
HIGH GRADE	4	16.0	14	33.33	9	21.43	45
NSC*	12		22		25		88
**TOTAL**	37		64		67		244

^*^NSC not subjected to this classification system and excluded from the % within breed for each grade

^**^ Includes all diagnosed MCTs in the sample population (*n* = 244; Patnaik’s = 225; Kiupel’s = 156; both systems = 137), regardless of breed

Benign soft tissue tumors were the second most common tumor type group, consisting in lipomas, fibromas, myxomas and fibrolipoma. Lipomas represented 74.8% of the total group, followed by fibromas (21.7%), myxomas and fibrolipoma. The two most common breeds affected were mixed-breed dog and Labrador retrievers. These tumors were most commonly seen on the forelimb, pectoral region and costal region (8.7%). However, most of cases displayed multicentric development. Female dogs had a higher frequency compared to males older dogs were mainly affected.

In this group, lipomas were the most discerned neoplasm and specific variants were observed. Similarly, to the main group, lipomas were commonly seen in mixed-breed dogs and Labrador retrievers. However, cocker spaniels (2.3% of all breeds, 4.3% occurrence within breed) and bassett hounds (2.33% of all breeds; 15.38% occurrence within breed) were also affected, with bassett hounds having the highest occurrence within breed. In descending order, the anatomical distribution was multicentric development, pectoral region and costal and forelimb region. Females (72.1%) were more affected and the highest frequency age interval was 8 to 12-years old. Fibromas most often occurred in the abdominal region and forelimb and breeds commonly affected were mixed-breed dogs, Labrador retrievers, poodles and Rottweilers with the last two having a higher breed-specific occurrence than the first two breeds. Females were also more affected, and the highest frequency age interval was 7 to 13-years-old.

Benign sebaceous tumors included sebaceous adenomas (64.6% within the group) and epitheliomas. Multicentric development was often found, along with neck and ear. This entity common concerned mixed-breed dogs, Labrador retrievers and cocker spaniels. Cocker spaniels recorded a high breed-specific occurrence in comparison to other breeds. For sebaceous adenoma, locations such as neck area and cranial region were also common. Sebaceous epithelioma (2.9% of all tumor types) was commonly found in the ears and facial region. Males were more affected than females while the opposite happened with sebaceous adenoma. In this group, both tumor types had a higher number of cases in older dogs.

Vascular tumors comprised 7.9% of all cutaneous tumor types and included hemangioma, hemangiosarcoma and lymphangioma. Tumors from this group were often found in hindlimb, followed by multicentric development and abdominal region. The most common breeds were mixed-breed dogs, Labrador retrievers, and boxers. However, Dogo Argentino (5.3%) had a 71.4% of breed-specific occurrence, higher than the previous breeds mentioned. Hemangiomas were the most frequent neoplasms, detected mainly in mixed-breed dogs, Labrador retrievers and boxers in the hindlimb, abdominal, dorsal and forelimb region. Hemangiosarcomas were often found in the perigenital area followed by multicentric development. Dogo Argentino presented the highest occurrence within breed in hemangiomas and hemangiosarcomas (42.9%; 28.6%, respectively).

Soft tissue sarcomas (STS) comprised fibrosarcoma, peripheral nerve sheath tumor, sarcomas NOS, perivascular wall tumors, synovial sarcoma, myxosarcomas and liposarcoma. STS were often diagnosed in mixed-breed dogs between 9 and 12 years-old dogs, being commonly found in the hindlimb and forelimb region. In this group, 54.4% of the lesions were benign and 45.6% were malignant. Peripheral nerve sheath tumors (43.3% of all STS) were mainly diagnosed in the extremities (forelimb and hindlimb) of mixed-breed dogs, displaying a higher number of cases in older dogs. A high number of perivascular wall tumor cases was also found in comparison to others diagnosis (22.2% of all STS), affecting predominantly older dogs of mixed breed and originating in the hindlimb and forelimb region..

Hepatoid gland tumors represented the sixth most common neoplasms in this case load. Common breeds affected were mixed-breed dog, Labradors Retriever and Estrela mountain dog, with the later presenting a higher breed-specific occurrence. Usual sites of development were the buttock area, followed by multicentric development and tail. These tumors had a higher tumor frequency in older and male dogs (86.4%), compared to female dogs (13.6%).

Hair follicle tumors included trichoepithelioma, trichoblastoma, infundibular keratinizing acanthoma, pilomatricoma, subungual keratoacanthoma, tricholemmoma and pawpad keratoma. For this group, the most common breeds were mixed breed, German shepherds and bassett hounds. Bassett hounds presented a higher breed-specific occurrence. These tumors were often detected in the hindlimb, dorsal region and neck (11.6%). Within this group, trichoepitheliomas were the most frequent entities, usually presenting multicentric distribution and affecting mixed-breeds, bassett hound, boxers and golden retrievers. Bassett hounds had the higher breed-specific occurrence in comparison to the other breeds. Trichoblastoma was the second most common histotype often located in the head region (facial, cranial region and ears). Cocker spaniels and German shepherds had a higher occurrence within breed.

Histioproliferative disorders constituted the ninth type of CT herein observed. This group consisted of histiocytomas and histiocytic sarcomas. For histiocytomas, the three most common breeds were mixed-breed dogs, boxers and French bulldogs. French bulldogs had the higher breed-specific occurrence. This tumor type was usually found in the ears and facial and pectoral region. Histiocytomas had a higher frequency in young dogs especially under 3-years-old (70.4% of all the cases), however 1-year-old dogs were most often affected (25.9%).

Epidermal tumors were the tenth most common CT type affecting mainly older dogs. By descending order: squamous cell carcinoma (SCC), papilloma, and basal cell carcinoma. Mixed breed dogs, Labradors Retrievers and Dogo Argentino were the most common breeds. Dogo Argentino had the highest breed-specific occurrence in comparison to the other two breeds. These tumors commonly displayed multicentric location and were often found in the forelimb and facial region. The most common breeds with SCC were mixed-breed dogs, Dogo Argentino and Labrador retriever, with Dogo Argentino presenting higher occurrence within breed of the total breeds mentioned. SCC regularly displayed multicentric development followed by facial, forepaw and hindlimb region. This tumor type frequently affected male dogs within the highest frequency age interval of 9 to 12-year-old. Three cases of SCC were subungual and all were diagnosed in male and older dogs.

Melanocytic tumors represented only 3.6% of all CT and comprised melanocytomas and melanomas. Melanocytomas occurred often in mixed-breed dogs (43.48%), dogue the Bordeaux and pinscher (8.7% each breed) with a breed-specific occurrence of 1.10%, 28.6% and 4.4%, respectively. These neoplasms presented multicentric development (21.7%) and often occur in the abdominal region (17.4%). Melanomas had a higher number of cases in Labrador retrievers (45.0%), mixed-breed dogs (15.0%) and golden retrievers (10.0%). Labrador retrievers and golden retrievers had a higher breed-specific occurrence (2.9%, 3.9%) in comparison to mixed-breed dogs (0.3%). This tumor type was diagnosed in hindpaw (20.0%), forepaw (15.0%) and as multicentric lesions (15.0%). Older dogs were more affected with melanocytic tumors.

The less common tumor types were plasmacytic tumors and apocrine gland tumors, musculoskeletal tumors, epithelial tumor NOS, lymphomas, round neoplasm NOS, mesenchymal neoplasm NOS, cutaneous neoplasm NOS, and mixed mesenchymal neoplasm.

### Binary logistic regression analysis: The influence of sex, age, and breed on the development of cutaneous tumors

In order to investigate the effect of sex, breed, and age in the development of cutaneous tumors, not discriminating malignancy, a binary logistic regression model was used [8, 18]. For the analysis of the three factors (age, sex, and breed) two models were performed: one consisting in comparing age, sex, and purebred vs mixed breed; and the other model discriminated all specific breeds. These results are represented in Table 4 and Table S2, respectively. In the first model, increased odds for developing cutaneous neoplasms as the patient’s age increased was detected (Table 4). Regarding sex, female dogs showed an increased odds in comparison to male dogs (crude OR = 2.99, 95% (2.51, 3.55); adj OR = 2.93, 95% (2.46, 3.49)). Purebred dogs demonstrated decreased odds for developing cutaneous tumors in comparison to mixed-breed dogs (crude OR = 0.63, 95% (0.53, 0.74); adj OR = 0.75, 95%, (0.62, 0.89)). In the second model, identical results were obtained for age and sex. (Table S2). However, two specific breeds, poodles and Yorkshire terriers, revealed increased odds in comparison to mixed breed dogs. Nine breeds presented decreased odds for developing CT in comparison to mixed breed dogs, namely bassett hound, boxer, cocker spaniel, Dogo Argentino, French bulldog, golden retriever, Labrador retriever, pit bull and pug (Table S2).

**Table 4:** Binary logistic regression analysis showing the association of cutaneous tumors development with age, sex, and purebreds vs mixed breed dogs

FACTOR	CRUDE OR (95%CI)	ADJ. OR (95%CI)	P (LR-TEST)	P (WALD'S TEST))
**AGE (QUARTIS)**			< 0.001	
0 – [0–8 YEARS OLD]*	1.00 (REF)	1.00 (REF)		
1 – [8–10 YEARS OLD]	2.03 (1.62,2.56)	1.97 (1.56,2.5)		< 0.001
2 – [10–12 YEARS OLD]	2.02 (1.61,2.54)	1.91 (1.5,2.42)		< 0.001
3 – [12–19 YEARS OLD]	2.05 (1.63,2.58)	1.89 (1.48,2.42)		< 0.001
**SEX**			< 0.001	
MALE*	1.00 (REF)	1.00 (REF)		
FEMALE	2.99 (2.51,3.55)	2.93 (2.46,3.49)		< 0.001
**PUREBRED VS MIXED**			< 0.001	
MIXED*	1.00 (REF)	1.00 (REF)		
PUREBRED	0.63 (0.53,0.74)	0.75 (0.62,0.89)		< 0.001

^*^REF, reference group

## Discussion

The skin is the largest organ in the body and accommodates populations of epithelial, mesenchymal, and local immune cells, which play an important role in homeostasis and protection against external factors. In comparison with previous studies performed in other European countries and in the United States [11, 18], and despite the fairly small sample size, this research provides insights regarding the most commonly diagnosed cutaneous tumor histotypes and their most probable anatomical sites of occurrence, patient ages and genders, as well as breeds in greater risk in this specific geographic region. Differences between current and preceding CCT epidemiological investigations may be attributed to methodological variations amongst studies (e.g., data and inclusion/exclusion criteria) [8, 10, 11, 18]. Thus, a prudent results interpretation is mandatory.

As in preceding studies, MCTs are one of the most common canine lesions. Due to their large prevalence and varied biological behavior, several histologic grading methods have been developed for defining cutaneous MCT prognosis [4, 10, 13, 17–19]. Similarly to previous reports, herein more than two-thirds of all the cutaneous MCT cases subjected to Kiupel’s grading system were classified as low grade and, less than one-third as high grade [17, 20, 21]. However, in contrast with other investigations and according with Patnaik classification system, in the present study both grade II (intermediately differentiated – 64,9%) and grade III (poorly differentiated – 22.2%) displayed higher frequency than grade I (well differentiated – 12.9%) [19, 22, 23]. Subcutaneous MCT were less frequent than the cutaneous variant. In agreement with several investigations, a high breed-occurrence was recorded in Labrador retrievers and boxers [11, 22, 24–27]. Boxers had a higher number of cases classified as Kiupel’s low grade and as Patnaik’s grade I and II. Conversely, a higher number of Patnaik’s grade II and III lesions were diagnosed in Labrador retrievers. But according with Kiupel’s system this difference was not observed, since a low number of cases was classified as high grade in Labrador retrievers. Nevertheless, about 40% of cutaneous MCTs diagnosed in this breed were not subjected to this classification, which can further influence and limit results inferences. These observations corroborated previous findings describing that boxers have predisposition to low grade MCTs and, hypothetically, Labrador retrievers to more aggressive MCT forms [24, 28–30]. Mixed-breed dogs had a higher proportion of grade II and III tumors (*n* = 55) and, therefore, presumably more aggressive tumors. However, with Kiupel's classification, mixed breed dogs presented mainly low-grade tumors. This disparity could be due to sampling, since a significant proportion of cases were not subjected to Kiupel's classification system (*n* = 225 with Patnaik vs *n* = 156 with Kiupel).

Fibromas are commonly reported in the limbs and head but herein, most were mainly observed in the abdominal region, limbs and dorsal region [27, 31]. Lipomas had a higher frequency in female dogs, reinforcing data that bitches have a predisposition for these tumors [32]. In terms of anatomical distribution, the majority of the cases presented a multicentric development however, pectoral, costal, and dorsal regions as well as limbs were also commonly affected as seen in previous studies [27, 31, 33, 34]. In accordance with other investigations, Labrador retrievers and cocker spaniels have an increased risk to develop lipomas [27, 35–39]. Despite not being suggested as a breed at risk, our results showed a high occurrence in bassett hounds. Since it is suggested that lipomas have an association with obesity/overweight, this might be one explanation for the highest occurrence in this breed, as bassett hounds are well known for having weight disorders [38, 39]. In our series and similarly to other studies, a small portion of lipomas was infiltrative [34, 40–42] and affected mainly the limbs (66.66%) of female dogs [27, 31].

Sebaceous tumors are frequently found in the facial, cranial, and cervical region with a high tendency for multicentric growth, and a notable occurrence in Labrador retrievers and cocker spaniels as also currently observed [27, 34].

Hemangiomas commonly occur in Labrador retrievers, boxers, Dogo Argentino, German shepherd and golden retrievers. In turn, hemangiosarcomas are often identified in Dogo Argentino, German shepherd and pit bull. Indeed, herein the relative proportion of Dogo Argentino with vascular tumors is higher than in any other breed. According to the literature, older, shorthaired and light skin dogs can have an increased risk for the development of these tumors which highlights the importance of prolonged solar exposure in the pathogenesis of these neoplasms. [27, 31, 43]. Additionally, it is important to note that the geographic region where the study took place is sunny, has a mild climate without excessive thermal amplitudes, being only marked by rainy and windy winters. It is described that limbs and abdominal region are usual sites for solar induced hemangiomas and dorsal regions for non-sun induced hemangiomas [31, 33, 44] and these evidences can also be applied for hemangiosarcomas [32]. In our series there were some cases observed in the abdominal area however, the most popular was the perigenital area along with multicentric development that also favors the theory of solar action in the trigger of these neoplasms.

As previous reported, large breed dogs are at increased risk for perivascular wall tumor [34, 45–49] and the two most common breeds were primarily mixed-breed, and secondly boxers [27, 31]. Similar findings were observed concerning peripheral nerve sheath tumors [31, 34, 50, 51].

As herein described and in agreement with other studies, hepatoid gland tumors remain the most common male canine lesions. In accordance with literature, males have a 5.6 times increased risk of developing hepatoid tumors in comparison to females [52, 53]. The evidence that androgen receptors (ARs) are present in all normal canine hepatoid tissues combined with the fact that males have higher levels of this hormone in circulation may justify the greater occurrence of these lesions in this gender. [52]. Additionally, the presence of ARs in perianal tumor tissue implies that derived tumors are hormone-dependent and thus, hormonal changes might influence tumor development. In terms of hepatoid carcinoma, Siberian Huskies presented a higher breed-specific occurrence [27, 34, 54–56]. Although the present work only focus in cutaneous tumors, this breed is described as having a 2.1 to 4.0 risk of developing testicular interstitial cell tumors, which are hormonally active tumors [57]. Furthermore, clinical evidences show that these testicular tumors are correlated with an increase in systemic androgens and with hepatoid tumors development [53, 57], factors that can contribute to the potential higher occurrence of hepatoid neoplasia in this breed.

Trichoblastomas were often found on the head and neck of male dogs [31, 34, 58] and, as reported, Cocker spaniels and mixed-breed dogs were commonly affected [31, 34]. As already reported, the bassett hounds included in this study also exhibited multicentric trichoepithelioma growths [27, 31, 34]. In addition, Golden retrievers were also frequently diagnosed with this tumor [34]. Infundibular keratinizing acanthomas were often detected in the dorsal, neck and hindlimb [31, 34] and German shepherds were amongst the mainly affected and are one of the breeds linked to the development of these tumors in various studies [27, 31]. Hair follicle tumors often affect animals with short and thick hair, which corresponds to the hair characteristics of the breeds described above.

Currently, histiocytomas were frequent in dogs with age interval less than 3 years old but specially, in puppies with ≤ 1-year-old [31, 34]. Most of the lesions were solitary and concentrated in head regions (facial, cranial regions and ear) and limb extremities which is also consistent with the literature [31, 34]. Boxers have a predisposition to the development of histiocytoma and as such, in this study it had a higher breed-specific occurrence [59].

Squamous cell carcinomas developed in mature and senior dogs and occurred primarily with a multicentric distribution but solitary lesions were also detected in the head, limbs and abdominal region [31, 34]. It is worth mentioning the diagnosis of three cases of subungual SCC, all affecting male dogs. This tumor, arising in this precise location has different prognosis in comparison to other skin regions [60]. Belluco et al. showed that canine digital SCC hardly ever metastasized but present a higher occurrence rate and multicentric growth [60]. In the present study, only one case of subungual SCC presented multicentric distribution, all the remaining cases were solitary masses [61]. Similar to our results, in which two of three dogs with subungual SCC were mixed-breed dogs and the third as a Labrador Retriever, these two breeds were among the most affected by this histotype in a prior study[62].

Canine melanocytomas can originate in any part of the body but often appear in the truncal area and occasionally, in the extremities [31, 63]. A great proportion of the melanocytomas herein analyzed had restricted locations however, the frequency of cases displaying multicentric development was higher than expected (21.7%). According with literature, sunlight may have a role in the development of canine melanomas in areas of the body with sun-exposed skin, such as the face and pinnae [27, 32, 64, 65]. However, in the present study the most prevalent sites were the limbs or head. Therefore, other etiologies such as consanguinity, trauma, chemicals exposure, hormones, and genetic predisposition must be taken into account [65].

A predisposition of Old English sheepdogs for apocrine adenomas is documented in the literature [34]. In our series there is a single case of this specific breed presenting this kind of lesion. Thus, although the breed-specific occurrence is high, we do not have enough data to support this previous suggestion. Our findings, together with prior studies, suggest a higher occurrence for plasmacytic tumors in male dogs compared to female dogs. Additionally, the great majority of our cases consisted in solitary lesions, often observed in the limbs and in the facial region, which also corroborates other works [66, 67].

Tumors with a low frequency (*n* < 5) in our database were not sufficient for a correct analysis therefore, they were not considered for comparison purposes nor for inferences related with their epidemiological behavior.

Despite using a different method for age assessment, our results are consistent with previous studies [8, 18] showing that older dogs are at increased odds for cutaneous tumors development. For gender, the results are varied, with some studies showing risk differences related to gender [18] while in others no differences were observed [8]. In our study, when evaluating sex as a factor for the development of cutaneous tumors, female dogs had a significantly increased OR compared to male dogs, which is in line with some investigations [18] and in contrast to most of them [1, 68–70]. In this case series, the reproductive status of most of the animals was unknown. Nevertheless, it is known that neutering can reduce the risk of tumor development of some malignancies, especially in what concerns to reproductive tract neoplasias [71, 72].

In our analysis purebred dogs showed a decreased odds in comparison to mixed breed dogs, differing from overall studies. Only two specific breeds appear to be at increased odds in comparison to mixed breed dogs, namely poodles and Yorkshire terrier. For poodles, these results are in concordance with previous studies [8] however, for Yorkshire terrier, to the best of our knowledge no similar results were documented. Besides that, some predisposed breeds showed a relatively lower odds for tumor development than mixed-breed dogs, such as bassett hound, boxer, cocker spaniel, Dogo Argentino, French bulldog, golden retriever, Labrador retriever, pit bull and pug [8].

These results, however, do not imply that the breeds found in this study with a decreased OR are not at risk at all for the development of cutaneous neoplastic lesions, since they do not represent the odds for the breed in general. These results only highlight that, in this specific region, several factors, such as breed preference, breeding practices and living conditions play an important role in the development of cutaneous neoplasia. Thus, caution in this interpretation is needed. This is the first study of this kind in Portugal, whose results may serve to elucidate and inform the veterinary community about the occurrence of these lesions and to assist clinicians in formulating their clinical judgments and preliminary differential diagnoses. As limitations, we emphasize the fact that the data included was collected from only one national laboratory, located specifically in the northern zone of the country and that the analyzed casuistic belongs to a specific period of time, so it may not be entirely representative of the country's global reality.

## Conclusions

In summary, the present paper describes the epidemiology of CCT in the animal data retrieved from ICBAS-LPV laboratory and demonstrates the relative frequency of the different tumors types in this particular population. The great majority of the cutaneous neoplastic lesions were benign. Mast cell tumor, benign soft tissue tumors, sebaceous tumors, vascular tumors, and soft tissue sarcomas were the most usual tumor types. The discrepancy between some already published results and the outcomes herein achieved might be the reflex of different etiologies and environmental factors that may play a role in the development of these neoplasms throughout the various geographical regions considered. Nevertheless, the epidemiological information obtained with this study can act as a basis or a beneficial reference for regional veterinarians to determine a preliminary diagnosis of CCT.

## Supplementary Information

**Additional file 1: Figure S1*.*** Age distribution according to tumor types

**Additional file 2:** **Table S1. **Number (n) and frequency (%) of the breeds from the Canine population in the database, 2014-2020 used in the calculations for occurrence within breed. **Table S2.** Binary logistic regression analysis showing the association of cutaneous tumors development with age. sex. and specific breed

## Data Availability

All data generated or analyzed during this study are included in this published article and its supplementary information files.

## Abbreviations

ARS: Androgen receptors
ER: Oestrogen receptors
CCT: Canine cutaneous tumors
CT: Cutaneous tumors
MCT: Mast cell tumor
OR: Odds ratio
STS: Soft tissue sarcomas
SCC: Squamous cell carcinoma
NOS: Not otherwise specified
NOC: Not otherwise classified
NSC: Not subjected to this classification system
REF: Reference group
