# Supplementary figures and images for: Retrospective study of canine cutaneous tumors submitted to a diagnostic pathology laboratory in Northern Portugal (2014–2020)
Source: Canine Med Genet. 2022 Feb 25;9:2. doi: 10.1186/s40575-022-00113-w (PMC8875941; doi:10.1186/s40575-022-00113-w)

**Figure S1** Age distribution according to tumor types


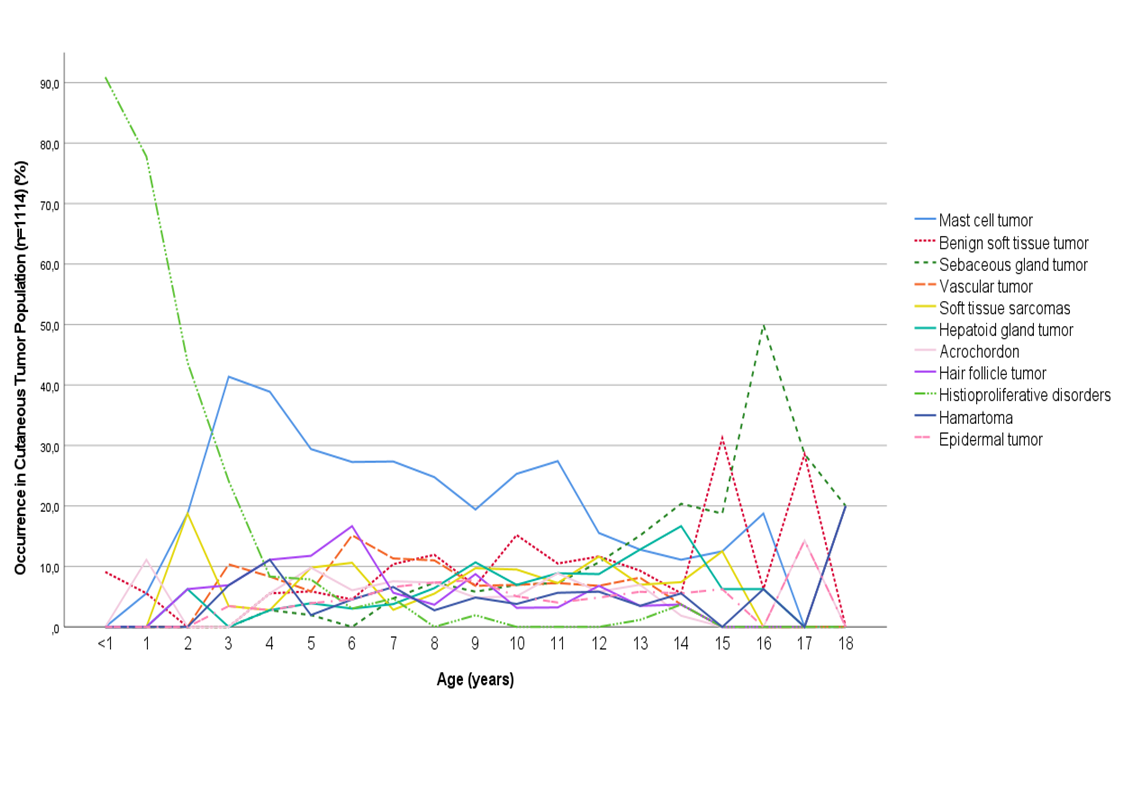

Supplement: Supplementary file 1 — Additional file 1: Figure S1. Age distribution according to tumor types [file 40575_2022_113_MOESM1_ESM.docx]
