# Supplementary material for: Retrospective study of canine cutaneous tumors submitted to a diagnostic pathology laboratory in Northern Portugal (2014–2020)
Source: Canine Med Genet. 2022 Feb 25;9:2. doi: 10.1186/s40575-022-00113-w (PMC8875941; doi:10.1186/s40575-022-00113-w)
Supplement: Supplementary file 2 — Additional file 2: Table S1. Number (n) and frequency (%) of the breeds from the Canine population in the database, 2014-2020 used in the calculations for occurrence within breed. Table S2. Binary logistic regression analysis showing the association of cutaneous tumors development with age. sex. and specific breed [file 40575_2022_113_MOESM2_ESM.docx]

**Table S1** Number (n) and frequency (%) of the breeds from the Canine population in the database, 2014-2020 used in the calculations for occurrence within breed

| Breeds | n | % |
| --- | --- | --- |
| Mixed | 908 | 39.6 |
| Labrador Retriever | 315 | 13.7 |
| Boxer | 136 | 5.9 |
| German Shepherd | 90 | 3.9 |
| Yorkshire Terrier | 75 | 3.3 |
| Poodle | 70 | 3.1 |
| Golden Retriever | 52 | 2.3 |
| Cocker Spaniel | 47 | 2.1 |
| Pinscher | 46 | 2.0 |
| French Bulldog | 41 | 1.8 |
| Beagle | 31 | 1.4 |
| Pit Bull | 28 | 1.2 |
| Rottweiler | 25 | 1.1 |
| Siberian Husky | 22 | 1.0 |
| Estrela Mountain Dog | 20 | .9 |
| Cocker | 17 | 0.7 |
| Dalmatian | 15 | 0.7 |
| Great Dane | 14 | 0.6 |
| Samoyed | 14 | 0.6 |
| Basset Hound | 13 | 0.6 |
| Doberman | 13 | 0.6 |
| West Highland White Terrier | 13 | 0.6 |
| Bouvier Bernois | 12 | 0.5 |
| Dachshund | 11 | 0.5 |
| Epagneul Breton | 10 | 0.4 |
| Jack Russell Terrier | 10 | 0.4 |
| Pug | 9 | 0.4 |
| Schnauzer | 9 | 0.4 |
| Bullmastiff | 8 | 0.3 |
| Chihuahua | 8 | 0.3 |
| German Shorthaired Pointer | 8 | 0.3 |
| Shitzu | 8 | 0.3 |
| Spitz | 8 | 0.3 |
| Bulldog | 7 | 0.3 |
| Castro Laboreiro Dog | 7 | 0.3 |
| Dogo Argentino | 7 | 0.3 |
| Dogue de Bordeaux | 7 | 0.3 |
| Shar-pei | 7 | 0.3 |
| American Staffordshire Terrier | 6 | 0.3 |
| Bull Terrier | 6 | 0.3 |
| Cane Corso | 6 | 0.3 |
| Pointer | 6 | 0.3 |
| Rhodesian Ridgeback | 6 | 0.3 |
| Warren Hound | 6 | 0.3 |
| Weimaraner | 6 | 0.3 |
| Border Collie | 5 | 0.2 |
| Chow-Chow | 5 | 0.2 |
| Maltese | 5 | 0.2 |
| Pekingese Dog | 5 | 0.2 |
| Portuguese Water Dog | 5 | 0.2 |
| St. Bernards | 5 | 0.2 |
| Barbado da Terceira | 4 | 0.2 |
| Bichon | 4 | 0.2 |
| Collie | 4 | 0.2 |
| English Setter | 4 | 0.2 |
| Irish Setter | 4 | 0.2 |
| Portuguese Mastiff | 4 | 0.2 |
| Portuguese Pointer | 4 | 0.2 |
| Belgian Shepherd | 3 | 0.1 |
| Flat Coated Retriever | 3 | 0.1 |
| Newfoundland Dog | 3 | 0.1 |
| Pomeranian Dog | 3 | 0.1 |
| Akita | 2 | 0.1 |
| American Cocker | 2 | 0.1 |
| Beauceron | 2 | 0.1 |
| English Mastiff | 2 | 0.1 |
| English Springer Spaniel | 2 | 0.1 |
| Fox Terrier | 2 | 0.1 |
| Greyhound | 2 | 0.1 |
| Staffordshire Bull Terrier | 2 | 0.1 |
| Transmontano Mastiff | 2 | 0.1 |
| Whippet | 2 | 0.1 |
| American Bulldog | 1 | 0.0 |
| American Pit Bull Terrier | 1 | 0.0 |
| Bernese Moutain Dog | 1 | 0.0 |
| Boerboel | 1 | 0.0 |
| Boston Terrier | 1 | 0.0 |
| Brazilian Mastiff | 1 | 0.0 |
| Briard dog | 1 | 0.0 |
| English Bulldog | 1 | 0.0 |
| Malinois | 1 | 0.0 |
| Neapolitan mastiff | 1 | 0.0 |
| Old English Sheepdog | 1 | 0.0 |
| Portuguese Sheepdog | 1 | 0.0 |
| Portuguese Warren Hound | 1 | 0.0 |
| Setter | 1 | 0.0 |
| Shetland Sheepdog | 1 | 0.0 |
| Staffordshire | 1 | 0.0 |
| Water dog | 1 | 0.0 |
| White Swiss Shepherd Dog | 1 | 0.0 |
| Total | 2291 | 100.0 |

**Table S2** Binary logistic regression analysis showing the association of cutaneous tumors development with age. sex. and specific breed

| Factor | crude OR (95%CI) | adj. OR (95%CI) | P (LR-test) | P (Wald's test)) |
| --- | --- | --- | --- | --- |
| Age (Quartis) |  |  | < 0.001 |  |
| 0 – [0-8 years old]* | 1.00 (REF) | 1.00 (REF) |  |  |
| 1 – [8-10 years old] | 2.03 (1.62.2.56) | 1.83 (1.41.2.36) |  | < 0.001 |
| 2 – [10-12 years old] | 2.02 (1.61.2.54) | 1.8 (1.39.2.34) |  | < 0.001 |
| 3 – [12-19 years old] | 2.05 (1.63.2.58) | 1.56 (1.2.2.04) |  | 0.001 |
| Sex |  |  | < 0.001 |  |
| MALE* | 1.00 (REF) | 1.00 (REF) |  |  |
| FEMALE | 2.99 (2.51.3.55) | 2.9 (2.4.3.51) |  | < 0.001 |
| Specific Breed |  |  | < 0.001 |  |
| mIXED bREED* | 1.00 (REF) | 1.00 (REF) |  |  |
| bassett hound | 0.12 (0.03.0.53) | 0.13(0.03.0.59) |  | 0.008 |
| French bulldog | 0.14 (0.05.0.42) | 0.25 (0.08.0.78) |  | 0.016 |
| pug | 0.16 (0.03.0.76) | 0.12 (0.03.0.59) |  | 0.009 |
| Dogo Argentino | 0.17 (0.06.0.52) | 0.15 (0.05.0.47) |  | 0.001 |
| pit bull | 0.29 (0.13.0.64) | 0.32 (0.14.0.74) |  | 0.007 |
| boxer | 0.41 (0.3.0.57) | 0.47 (0.34.0.67) |  | < 0.001 |
| golden retriever | 0.41 (0.24.0.7) | 0.55 (0.31.0.97) |  | 0.038 |
| Labrador retriever | 0.47(0.36.0.6) | 0.54 (0.41.0.71) |  | < 0.001 |
| cocker spaniel | 0.56 (0.35.0.9) | 0.58 (0.35.0.95) |  | 0.03 |
| Yorkshire terrier | 1.81 (1.04.3.14) | 1.86 (1.05.3.3) |  | 0.033 |
| poodle | 3.58 (2.03.6.29) | 3.27 (1.84.5.83) |  | < 0.001 |

**REF*. reference group
